# Scaffolding for Repair: Understanding Molecular Functions of the SMC5/6 Complex

**DOI:** 10.3390/genes9010036

**Published:** 2018-01-12

**Authors:** Mariana Diaz, Ales Pecinka

**Affiliations:** 1 Institute of Experimental Botany of the Czech Academy of Sciences (IEB), Centre of the Region Haná for Biotechnological and Agricultural Research, Šlechtitelů 31, 77900 Olomouc-Holice, Czech Republic; 2 Max Planck Institute for Plant Breeding Research (MPIPZ), Carl-von-Linné-Weg 10, 50829 Cologne, Germany; diaz@mpipz.mpg.de

**Keywords:** SMC5/6, genome stability, DNA damage repair, Structural maintenance of chromosomes, chromatin, chromosomes

## Abstract

Chromosome organization, dynamics and stability are required for successful passage through cellular generations and transmission of genetic information to offspring. The key components involved are Structural maintenance of chromosomes (SMC) complexes. Cohesin complex ensures proper chromatid alignment, condensin complex chromosome condensation and the SMC5/6 complex is specialized in the maintenance of genome stability. Here we summarize recent knowledge on the composition and molecular functions of SMC5/6 complex. SMC5/6 complex was originally identified based on the sensitivity of its mutants to genotoxic stress but there is increasing number of studies demonstrating its roles in the control of DNA replication, sister chromatid resolution and genomic location-dependent promotion or suppression of homologous recombination. Some of these functions appear to be due to a very dynamic interaction with cohesin or other repair complexes. Studies in Arabidopsis indicate that, besides its canonical function in repair of damaged DNA, the SMC5/6 complex plays important roles in regulating plant development, abiotic stress responses, suppression of autoimmune responses and sexual reproduction.

## 1. Introduction 

The eukaryotic nuclear genome is organized into linear chromosomes. Chromosomal DNA is wrapped around histone octamers forming nucleosomes. Nucleosomes are the primary chromatin units, which are folded into chromatin fibers and the fibers into domains of different density and accessibility [1,2]. Chromosome and chromatin stability is challenged by endogenous factors including free radicals, replication errors and topological stress [3]. Exogenous damage is exerted by adverse environmental conditions such as UV radiation, oxidative stress and chemical pollutants [4]. These (and other) factors challenge genome stability by a wide range of toxic effects including base oxidation, alkylation, DNA single and double strand breaks (SSBs and DSBs) and formation of non-native bonds within and/or between DNA strands [5]. Unrepaired or misrepaired lesions result in mutations, which compromise gene functionality, cause loss/gain of genetic information and induce chromosome instability. This problem may be particularly pronounced in obligatory phototrophic sessile organisms such as plants, which are exposed to challenging environmental conditions without possibility for escape [6,7].

**Figure 1:**
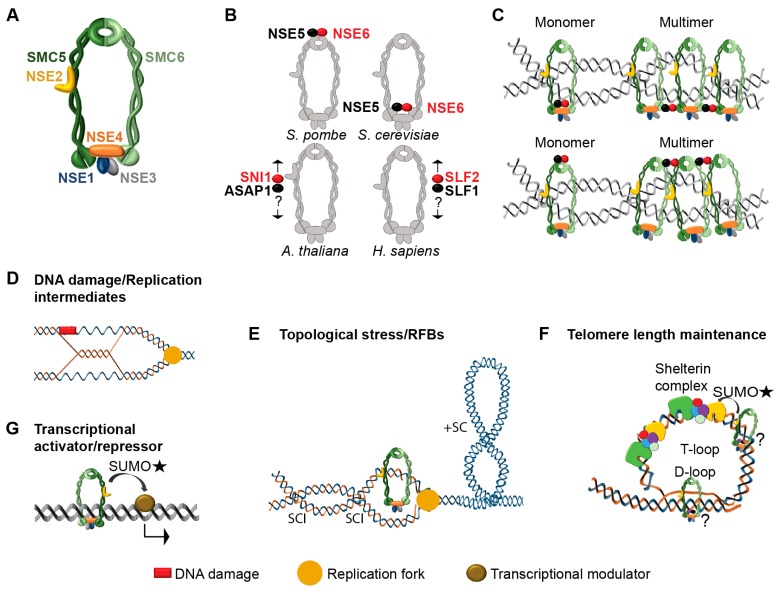
Structural maintenance of chromosomes (SMC) 5/6 complex composition and functions. (**A**) Consensual model of SMC5/6 complex without and (**B**) with species-specific positions of NON-SMC ELEMENT (NSE) 5(-like) and NSE6(-like) subunits in *Schizosaccharomyces pombe*, *Saccharomyces cerevisiae*, *Arabidopsis thaliana* and *Homo sapiens*. (**C**) Hypothetical function of NSE5-NSE6 dimer in multimerizing SMC5/6 complexes via their heads (top) or hinges (bottom). (**D**) Replication intermediate structure bypassing DNA damage site (red square). (**E**) Topological stress occurring during DNA replication and at replication fork barriers (RFBs) represented by the positive supercoil (+SC) ahead of the replication fork and sister chromatid intertwining (SCIs) between the nascent chromatids. (**F**) Role of SMC5/6 complex in telomere length maintenance. (**G**) Speculative model for SUMOylation of transcriptional modulations by SMC5/6 complex. Note that the position of SMC5/6 complex in images (**C**), (**E**), (**F**) and (**G**) is only speculative.

Structural maintenance of chromosomes (SMC) complexes are the key regulators of chromosome dynamics, structure and function in eukaryotes (reviewed in [8,9,10,11,12]). They operate from the scale of whole chromosomes in chromosome segregation to few base pairs in DNA damage repair. The core subunits of SMC complexes are SMC proteins, which are large polypeptides (1000–1300 amino acids) containing Walker A and Walker B motifs at their N- and C-terminal globular domains. The primary step towards functional SMC protein is folding at the hinge domain and coiling of the arms. This brings the C- and N-terminal globular domains together and constitutes heads with ATP-dependent DNA binding activity [13]. The most characterized SMC complex is cohesin (containing SMC1 and SMC3). It controls dynamics of sister chromatid cohesion and thus affects chromosome segregation, meiotic recombination and DNA damage repair (reviewed in [9,10,11]). Condensin complex (containing SMC2 and SMC4) plays a pivotal role in chromosome folding and condensation during interphase and nuclear division. Finally, the third complex consisting of SMC5 and SMC6 heterodimer backbone, called SMC5/6, is famous for its role in maintaining genome stability [8]). Beside the SMC5 and SMC6, this complex contains six additional NON-SMC ELEMENT (NSE) subunits (Figure 1A,B and Table 1) and the whole complex is organized into three sub-complexes: NSE2-SMC5-SMC6, NSE1-NSE3-NSE4 and NSE5-NSE6 acting as specialized functional modules [14,15,16]. In spite of increasing number of studies, the functions of SMC5/6 complex still remain relatively poorly understood. To foster this research, we provide an overview on the current understanding of SMC5/6 complex functions.

## 2. Architecture of SMC5/6 Complex

### 2.1. NSE1-NSE3-NSE4 Subcomplex

NSE1-NSE3-NSE4 trimer is a highly conserved part of the SMC5/6 complex responsible for binding DNA and bridging SMC heads. NSE1 contains a RING-like domain necessary for the NSE1-NSE3-NSE4 trimer formation and recruitment of NSE4 and SMC5 to the sites of DNA damage [17,18,19,20]. Mutations in the RING-like domain lead to DNA damage hypersensitivity and full deletion of *NSE1* is lethal in *Saccharomyces cerevisiae*, *Schizosaccharomyces pombe* and Arabidopsis [17,18,21]. In multiple organisms, it was shown that NSE1 interacts with the N-terminus of NSE3 subunit and strengthens its binding to dsDNA [15,16,20,21,22,23]. 

NSE3 occurs as single copy gene in fungi and plants but has homology to *MELANOMA ANTIGEN GENE* (*MAGE)* family with over 60 members in humans. MAGEs interact in vitro with E3 RING-type ubiquitin ligases. However, only MAGE-G1 and MAGE-F1 have been found to associate with NSE1 and only MAGE-G1 co-immunoprecipitated with the SMC5/6 holocomplex in human cells [19,24,25]. MAGEs are aberrantly expressed in a wide variety of cancer types and play a critical role in tumorigenesis [26,27]. The presence of MAGE-G1 and UBIQUITIN CONJUGATING ENZYME H 13 (UBCH13) and METHYL METHANE SULFONATE SENSITIVE 2 (MMS2) significantly enhances NSE1 E3 ubiquitin ligase activity [24]. 

The C-terminal domain of NSE3 interacts with NSE4 [22]. NSE4 is a structural protein containing a winged helix motif, which forms a RING-like structure through interaction with SMC proteins [16,28]. NSE4 is an essential protein and its functions include: interaction between NSE1-NSE3-NSE4 sub-complex and SMC5 as shown in *S. pombe* [15] and bridging SMC5 and SMC6 heads as found in *S. cerevisiae* [16]. 

### 2.2. NSE2-SMC5-SMC6 Sub-Complex 

NSE2-SMC5-SMC6 represents the core sub-complex, which serves as a central scaffold. Via NSE2/MMS1 enzymatic activity it most likely regulates dynamics of the whole complex at its target sites. NSE2/MMS21 was initially identified via genetic screen as hypersensitive to methyl methane sulfonate, X-rays and UV radiation in budding yeast [29] and was associated with SMC5/6 complex only about two decades later [30]. NSE2/MMS21 is covalently bound to the SMC5 protein (Figure 1A) and this association appears to be conserved in fungi, animals and plants [14,15,31,32]. NSE2/MMS21 contains a putative Protein Inhibitor of Activated STAT-Signal Transducer and Activator of Transcription (SIZ/PIAS) RING domain characteristic of Small Ubiquitin-like Modifier (SUMO) ligase [30]. In general, SUMO modification is involved in various cellular processes, such as nuclear-cytosolic transport, transcriptional regulation, apoptosis, protein stability, response to stress and progression through the cell cycle [33,34]. However, which of these (and potentially other) processes can be assigned to NSE2/MMS21 is largely unknown. In vitro studies revealed that NSE2/MMS21 adds SUMO modifications to (SUMOylates) numerous proteins, some in a species-specific manner. So far identified NSE2/MMS21 targets include SMC6, NSE3 and NSE4 in fission yeast; SMC5 and KU70 in budding yeast; SMC6, cohesin subunits SA2 and SCC1, Translin associated factor-X (TRAX) and several members of the SHELTERIN/TELOSOME complex in humans [20,30,32,35,36,37,38]. Surprisingly, in fungi, animals and plants NSE2 SUMOylates itself at the C-terminal region and thus most likely auto-regulates its own function. Catalytically dead human NSE2/MMS21 is not able to alleviate hypersensitivity to DNA damage, suggesting that NSE2/MMS21 SUMO ligase activity is required for proper cellular response to DNA damage [32]. Similarly to yeast and human, *A. thaliana* NSE2/MMS21/HPY2 (HIGH PLOIDY 2) was auto-SUMOylated in in vitro experiments and this activity could be abolished by H180A substitution in the SIZ/PIAS-RING domain [39,40]. This suggests that SIZ/PIAS-RING is crucial for the catalytic function of the protein. Though important, NSE2/MMS21 is not essential in Arabidopsis. The mutants are viable but have strong developmental phenotypes including small growth, deformed leaves, stem fasciations and are partially sterile and produce reduced number of seeds.

### 2.3. NSE5-NSE6 Sub-Complex 

The NSE5-NSE6 sub-complex is most likely responsible for loading, localization or multimerization of SMC5/6 complex (Figure 1B,C) and represents its evolutionarily non-conserved part. This sub-complex was identified via proteomic experiments using fungi, plants and *Xenopus laevis* egg extracts as a pair of unknown SMC5/6 associated proteins (Table 1); these include YML023c (alias NSE5) and KRE29 in budding yeast [30] and NSE5 and NSE6 in fission yeast [41], ARABIDOPSIS SNI ASSOCIATED PROTEIN 1 (ASAP1) and SUPPRESSOR OF NPR1, INDUCIBLE 1 (SNI1) in Arabidopsis [42] and SMC5-SMC6 complex localization factors 1 and 2 (SLF1 and SLF2) in vertebrates [43]. KRE29, NSE6, SNI1 and SLF2 contain armadillo (ARM) repeats [44], which are supposed to form a superhelix of α-helices resulting in a spiral structure. 3D modeling suggested that all these factors have a highly similar protein structure, where several essential residues of the Armadillo (ARM)-repeats create a binding surface not apparent from the linear sequence [41,42]. Their interaction partners YML023c, SLF1 and ASAP1 are considered as the putative functional orthologues of NSE5. Beside little conserved protein sequence, NSE5 and NSE6 differ also with respect to their position in the complex. In budding yeast NSE6 and NSE5 bind to the hinges of SMC5 and SMC6 [15], while in fission yeast they bind to SMC5 and SMC6 heads, without directly interacting with the NSE1-NSE3-NSE4 trimer (Figure 1B) [16,41]. Location of SNI1 and ASAP1 in Arabidopsis and SLF1 and SLF2 in vertebrates remains unknown (Figure 1B). The function of NSE5-NSE6 is unclear but an earlier study [16] proposed that this sub-complex could mediate SMC5/6 complex multimerization (Figure 1C). In vertebrates, SLF1-SLF2 subcomplex mediates interaction of SMC5/6 with RAD18 E3 ubiquitin protein-ligase during the process of DNA damage repair at stalled replication forks [43]. Both NSE5 and NSE6 were shown to be essential in budding yeast but not in fission yeast. In *A. thaliana*, loss of *SNI1* function leads to smaller and poorly looking plants with strongly reduced fertility. Homozygous *ASAP1* mutant plants are not able to develop beyond the cotyledon stage and die [42].

## 3. SMC5/6 Complex Molecular Functions

### 3.1. DNA Damage Repair

Repair of damaged DNA represents the canonical function of SMC5/6 complex and multiple complex subunits were identified in genetic screens based on mutant hyper-sensitivity to genotoxic stress [35,43,45,46,47,48]. Because the role of the SMC5/6 complex in fungal and animal DNA damage repair was summarized in several recent reviews (See [11,49,50,51]), we will focus mainly on the plant data in this section. 

Arabidopsis *SMC6B* and *NSE2*/*MMS21*/*HPY2* (high polidy 2) mutants show moderate hypersensitivity to UV, X-rays and mitomycin C (MMC) and strong hypersensitivity to MMS and zebularine [39,47,52,53,54]. While genotoxic effects of most of these treatments are generally well understood [55], effects of zebularine remain less clear. Our group found that besides its (relatively weak) DNA demethylating effects [53,56,57], it acts as a potent inducer of enzymatic DNA-protein crosslinks [58]. Collectively, the DNA damage assays indicate that the SMC5/6 complex participates in (post-)replicative repair of mainly complex or bulky lesions (Figure 1D) and has only a negligible role in non-homologous end joining repair of DNA double strand breaks (DSBs) in Arabidopsis. 

In animals and fungi, both cohesin and SMC5/6 complexes are recruited to DSB sites [38,59,60,61,62]. Initial observations in human cells revealed that the SMC5/6 complex recruits cohesin, facilitating repair by homologous recombination (HR) and this recruitment was dependent on NSE2/MMS21 mediated SUMOylation [38]. Recent study using *Xenopus laevis* eggs and human cells revealed that the recruitment of SMC5/6 to the sites of DNA damage in vertebrates is dependent on RAD18 and newly identified NSE5- and NSE6-like subunits SLF1 and SLF2 [43]. In contrast, the yeast SMC5/6 complex requires loading to chromosomes via the SCC1 subunit of cohesin complex [59,60,63]. Hence, loading of the SMC5/6 complex may be process- and/or species-specific. Data using flow-sorted Arabidopsis G2 nuclei revealed an SMC5/6-dependent increase in sister chromatid alignment upon induction of DNA damage, which depends on correct S phase-mediated cohesion [52]. 

Using transgenic reporter systems it was shown that Arabidopsis *SMC6A*, *SMC6B* and *NSE2*/*MMS21* mutants have reduced frequency of HR under control conditions [47,52,54]. Upon genotoxic treatments, wild-type and also *SMC6A* and *SMC6B* mutants (*NSE2*/*MMS21* was not tested) showed similar fold-increase but the total number of HR events still remained much lower in the mutants. In contrast, expression of *SMC6B* under the control of a strong constitutive viral promoter doubled HR frequency [47,64]. This indicates that the plant SMC5/6 complex functions as positive regulator of HR in a regulatory network, where several pathways compete for processing lesions by different repair mechanisms. This observation is consistent with the fungal and animal models [18,38,62,65]. 

Two evolutionary conserved kinases ATAXIA TELANGIECTASIA MUTATED (ATM) and ATM AND RAD3-RELATED (ATR) are involved in signaling presence of DNA strand breaks and single stranded DNA (typically at stalled replication forks), respectively, within the HR pathway [66,67,68]. Processing of spontaneous damages (presumably induced by DNA replication) is controlled by ATR, while both kinases are involved in signalling the presence of zebularine-induced DNA damage [42,53]. Whether the Arabidopsis SMC5/6 complex is directly phosphorylated by ATM and/or ATR remains unknown. A recent study analyzing phosphoproteomic targets of ATM and ATR did not reveal any SMC5/6 members [69]. However, this could be due to the treatment with gamma-radiation producing mainly DSBs, i.e., substrate which is not a typical target of SMC5/6 complex-mediated repair in Arabidopsis. Alternatively, SMC5/6 complex members could be activated at transcriptional level. We consider this scenario less likely because none of the complex subunits was detected up- or down-regulated in genome-wide studies using wild-type and ATM and ATR mutant plants exposed to variety of DNA damaging treatments [53,68,70].

Homology based repair is particularly challenging in tandemly repeated genome regions. High similarity of individual repeat units increases the risk of HR between ectopic copies, which can lead to loss of genetic information [71,72]. Data from yeasts and animals suggest that the SMC5/6 complex is recruited to replication fork barriers (RFBs) in rDNA and telomeres during G2/M and controls HR (Figure 1E,F) [8,62,73,74,75]. Presence of the SMC5/6 complex at rDNA loci (and telomeres) reduces activity of the recombination proteins like RAD51 [76,77], while its loss is accompanied by formation of RAD52 foci, indicative of error-prone repair, increased frequency of holiday junctions, HR and chromosomal rearrangements [73,78]. An interesting mechanism reducing the risk of ectopic recombination at repetitive DNA was described in insects and fungi [76,77,78]. Here, SMC5/6 complex interaction with heterochomatin protein 1 (HP1) blocks HR in heterochromatin until its expansion and relocation of damage sites into euchromatic nuclear space poor in repetitive DNA. SMC5/6 dependent heterochromatin remodeling upon DNA damage has not been observed in plants so far but one study showed that the kinetics of DSB repair is slower in the *SMC6B* mutant [79]. This may indicate that the SMC5/6 complex affects repair kinetics in plants but whether this is accompanied with heterochromatin relaxation needs to be analyzed. 

### 3.2. Removal of Replication-Derived Toxic Structures

Assisting DNA replication machinery, removal of toxic replication structures and relief from DNA topological stress represent potentially highly conserved but only recently discovered SMC5/6 functions. Hypersensitivity of SMC5/6 mutants to the replication blocking agents, such as hydroxyurea (HU) and MMS, led to assumption that the complex may be involved in detoxifying toxic structures arising during DNA replication [80]. A recent study in budding yeast revealed that SMC5/6 complex functions are essential during (late) G2 phase but not in the other cell cycle stages including S-phase, under non-damaging conditions [75]. Absence of SMC5/6 during S-phase allows normal replication initiation and fork speed, suggesting that the SMC5/6 function is post-replicative [75]. To date, two major post-replicative functions of SMC5/6 complex have been described: (i) removal of DNA supercoils and sister chromatid intertwining (SCIs) and (ii) resolving toxic DNA replication intermediates (Figure 1D,E).

Progressive separation of the parental DNA strands by replication machinery leads to the accumulation of positively supercoiled DNA ahead of the replication fork and formation of SCIs, i.e., coiled dsDNA strands, behind the fork (Figure 1E). Both structures are problematic as they cause topological stress and hinder sister chromatid separation during mitosis, respectively and therefore need to be removed in order to allow normal cellular functions [81]. Experiments in budding yeast showed that DNA supercoils are resolved by the coordinated actions of type I TOPOISOMERASE 1 (TOP1) and type II TOPOISOMERASE 2 (TOP2), while SCIs are removed by the activity of TOP2 as shown in budding yeast [82]. Recently also the SMC5/6 complex was found to play a role in removal of DNA supercoils and formation of SCIs in *S. cerevisiae* [60,83]. It is assumed that SMC5/6 facilitates fork rotation by sequestering nascent SCIs that form behind the replication machinery, thus decreasing the level of replication-induced supercoiling [60,83]. The SMC5/6 complex is loaded to the sites of DNA topological stress by the cohesin complex during S-phase as indicated by the absence of chromosome bound SMC5/6 in cohesin mutant *scc1* but loss of SMC5/6 function does not affect cohesin localization [60]. Based on the experiments with circular DNA molecules, it was suggested that the SMC5/6 complex and TOP2 function as ATP-dependent DNA linkers, which facilitate intermolecular interaction of DNA molecules through their topological entrapment [13]. In addition, TOP2 causes SMC5/6 to dissociate from chromosome arms under non-stress conditions [60,83], possibly by efficient removal of SCIs, upon which the presence of SMC5/6 is no longer required. Depletion of human SMC5 and SMC6 results in abnormal distribution of TOPOIIα, a homolog of the yeast TOP2, which probably leads to accumulation and/or abnormal distribution of SCIs and aberrant chromosome segregation [84]. In budding yeast, SMC5/6 may oppose the SCI stabilizing activity of the cohesin complex in the absence of TOP2 activity and thus allow easier passive sister chromatid resolution at the end of chromosomes [60]. This suggests that the SMC5/6 complex controls the TOP2-independent SCI resolution pathway. This model is based on budding yeast where sister chromatids remain paired with each other after DNA replication [85]. In Arabidopsis, where centromeres and telomeres show the highest degree of cohesion, in spite of a generally low degree of sister chromatid association during interphase [86], the SMC5/6 complex activity may be stimulated on demand after e.g., occurrence of DNA damage [52].

Another important SMC5/6 function linked to the post-replicative phase is a rescue of the collapsed replication forks and repair of the replication-derived toxic HR intermediates [75,80,87]. These are typically represented by X-shaped holiday junction structures formed during template switch in HR events (Figure 1D). They arise during bypass synthesis, when DNA polymerase encounters a block during DNA synthesis, switches the template to the newly replicated strand and returns to the original template after the damage. It is known that HR intermediates are repaired synergistically by the SMC5/6 complex and the STR complex, which consists of RECQ type helicase SLOW GROWTH SUPPRESSOR 1 (SGS1), type I TOPOISOMERASE 3 (TOP3) and RECQ-MEDIATED GENOME INSTABILITY PROTEIN 1 (RMI1) containing domain of unknown function in budding yeast [80,88,89,90]. The SMC5/6 complex associates to SGS1 and SUMOylates the STR complex, which decreases the presence of recombination structures [80,89]. The resolution of branched structures seems to be dependent on the SUMOylation ability of NSE2/MMS21, as the SGS1 mutants, impaired in recognition of SUMOylated SMC5/6 complex, exhibited unprocessed holiday junctions at damaged replication forks, increased exchange frequencies between double helices during double-strand break repair and severe impairment in DNA end resection [87,88]. Furthermore, there is an alternative (non-canonical) HR intermediate resolution pathway represented by MUTATOR PHENOTYPE 1 (MPH1), MMS2 and the SHU complex in budding yeast. It was proposed that the SMC5/6 complex acts antagonistically to MPH1, in a pathway distinct from that of SGS1, preventing accumulation of toxic intermediate structures [91,92].

## 4. Plant-Specific SMC5/6 Complex Functions

SMC5/6 complex controls number of processes, which are unique to higher plants and we will provide their overview in this section. Many of these phenotypes appear to be critical for successful plant development and affect also economically important traits such as yield or stress resistance. However, for many plant SMC5/6 mutant phenotypes it cannot be currently unambiguously decided whether they are caused by the lack of complex’ DNA damage repair functions or other activities.

**Figure 2:**
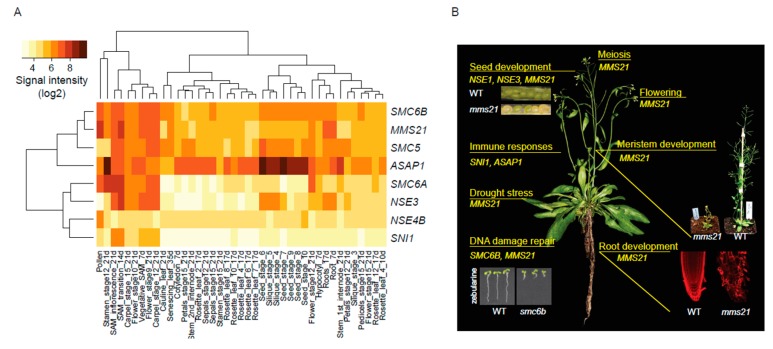
Structural maintenance of chromosomes (SMC) 5/6 complex in plants. (**A**) Log2 mRNA intensity values of genes encoding SMC5/6 complex in 49 Arabidopsis developmental stages. The primary ATH1 expression array data were derived from AtGenExpress dataset [93]. Please note that the *NSE1* and *NSE4A* are missing on ATH1 array. (**B**) Overview of SMC5/6 complex functions in Arabidopsis.

Plant SMC5/6 complex includes six evolutionarily conserved and two plant-specific (ASAP1 and SNI1) SMC5/6 subunits (Table 2). In spite of frequent polyploidization events during the evolution of seed plants, most subunits are represented by a single copy gene in the extant species. The only exception, is *NSE4*, which is represented by two or more copies in almost all analyzed seed plants (Table 2; note that the two SMC6 copies found in *A. thaliana* represent *Brassicaceae* family specific duplication event and are not found in other groups of vascular plants). Functional consequences of the NSE4 duplications remain unknown but transcriptional data from tomato and Arabidopsis suggest that some NSE4 copies are expressed only in specific developmental stages [52] (Table 2). By analysis of publicly available ATH1 expression microarray data [93] we show that Arabidopsis SMC5/6 complex subunits are expressed mainly in dividing tissues (Figure 2A; note that *NSE1* and *NSE4A* are missing on ATH1 chip). There were relatively strong differences between subunits and the strongest signals were found for *SMC5*, *SMC6B*, *NSE2*/*MMS21* and *ASAP1*. The strong signal for *ASAP1* contrasted with the low signal for its interaction partner *SNI1* in most tissues, possibly indicating different mRNA stability or mobility of both SMC5/6 members.

### 4.1. Developmental Regulator 

Multiple studies showed that the SMC5/6 complex regulates specific developmental processes including e.g., meristem and stem cell niche size, flowering time, meiosis, gametophyte and seed development in Arabidopsis [21,39,40,95,96,97]. The unifying theme of the affected tissues and biological processes is that they contain a high proportion of replicating nuclei and rapidly dividing cells, which could be associated with higher amounts of naturally occurring DNA damage and/or topological stress (Figure 1D,E). Moreover, some of these tissues represent germline cells, which appear to be under a strict control concerning genome and epigenome stability in plants [6,56,98,99,100].

Most developmental phenotypes controlled by SMC5/6 complex are described for *NSE2*/*MMS21*, which is (together with *SNI1*) the only non-duplicated subunit producing viable homozygous mutants. Arabidopsis *NSE2*/*MMS21* mutants (Figure 2B) were identified based on the short roots with increased nuclear endoploidy (therefore named as *HIGH POIDY 2* and abbreviated as *HPY2*), abnormally developed shoots with small leaves, irregular phylotaxy, occasional fasciations and partial sterility [39,40]. Cells within *NSE2*/*MMS21* mutant root apical meristems are disorganized and display an increased frequency of cell death. Molecular and genetic studies in Arabidopsis revealed that NSE2/MMS21 promote G1/S and G2/M transitions by destabilizing E2Fa/DPa transcription factor complex and promoting CyclinB1;1, respectively [39,101]. In parallel, NSE2/MMS21 affects other pathways during root development. The NSE2/MMS21 mutants show reduced response to exogenous cytokinin and down-regulation of transcription factors from cytokinin-induced arabidipsis response regulators (ARR) family [40]. There is also misregulation of stem cell niche-defining transcription factors [31] and recent study revealed that NSE2/MMS21 activity is required for high levels of BRAHMA chromatin remodelling factor and thus normal root development [102]. The phenotypes of *NSE2*/*MMS21* mutants are strengthened by application of exogenous DNA damaging factors, suggesting that inability to process particular types of toxic DNA structures represents another challenge [31].

Recently, NSE2/MMS21 was identified as floral repressor [96]. The *MMS21* mutant flowered earlier under both long and short day conditions, it had reduced amount of transcript and protein of the key floral repressor FLOWERING LOCUS C (FLC) and an increased transcript amount of the floral inducers SUPPRESSOR OF CONSTANS (SOC1) and flowering locus t (FT). FLC is the direct upstream regulator of FT, which then regulates SOC1 [103]. This indicates that the SMC5/6 complex promotes FLC transcription. This could occur via interaction or competition with Polycomb Repressive Complexes and/or LIKE-HETEROCHROMATIN PROTEIN 1 (LHP1), which are important modulators of FLC activity [104,105,106]. Besides altering FLC transcription, NSE2/MMS21 also SUMOylates FLC protein. We speculate that such activity could take place when FLC binds to its target gene FT and possibly alter FLC activity or stability (Figure 1G). Collectively, this suggests that NSE2/MMS21 prevents precocious flowering in Arabidopsis.

Once the decision to flower is reached, plants undergo a series of complex developmental events including production of gametes and seeds. The SMC5/6 complex plays critical role during multiple stages of generative development. *NSE2*/*MMS21* mutants showed lagging chromosomes and occasional anaphase bridges at meiotic metaphase I, indicating genome instability in male meiosis [97]. In addition, several transcripts for meiotic genes related to chromosome maintenance and recombination were altered in *NSE2*/*MMS21* mutants [97]. At the end of meiosis, *NSE2*/*MMS21* mutant plants developed not only tetrads but also dyads with large nuclei, which produced a smaller number of pollen, with poor germination and abnormal tube growth. Although, NSE2/MMS21 activity is required for successful male gametogenesis, the role of whole SMC5/6 complex in this process is far from being understood.

Fully developed micro- and mega-gametophytes, represented by pollen grains and ovules with mature embryo sac, respectively, allow double fertilization of egg cell and central cell and give rise to seeds. Seeds are important propagation units and source of nutrition for humans [107,108]. There is accumulating evidence that the SMC5/6 complex plays key role in seed development (Figure 2B). Homozygous mutants in multiple complex subunits: *SMC5* (alias *EMBRYO DEFECTIVE 2782*), *NSE1* (alias *EMBRYO DEFECTIVE 1379*), *NSE3* and *SMC6A SMC6B* double mutant do not produce viable seeds [21,42,52,109]. However, *NSE2*/*MMS21* and partially complemented *NSE1* and *NSE3* homozygous mutants produce 25% to 50% of aberrantly developed seeds and the viability of wild-type-like seeds is reduced [21,97]. Unhealthy seeds contained typically poorly developed, early stage-arrested, embryo and over-proliferated endosperm [21]. Although the mechanism of SMC5/6 complex involvement in seed development is currently unknown, its similarity with the *TITAN* seed phenotypes of cohesin and condensin mutants [110,111] makes it tempting to speculate that the underlying mechanism arises via combinatorial action of cohesin and SMC5/6 complexes [60] (see chapter 3.2. Removal of replication-derived toxic structures).

### 4.2. Modulator of Abiotic Stress Responses

It was reported that Arabidopsis *NSE2*/*MMS21* mutants show improved resistance to drought, while *NSE2*/*MMS21* over-expressors are drought hypersensitive [112]. NSE2/MMS21 works as a negative regulator of proline biosynthesis and drought tolerance is associated with higher proline concentrations, which could explain, at least in part, the phenotype observed. One of the responses to drought stress is abscisic acid (ABA) accumulation. NSE2/MMS21 expression is reduced upon ABA treatment. Mutations in *NSE2*/*MMS21* lead to upregulation of ABA-mediated stress responsive genes and to hypersensitivity to ABA, as indicated by stomatal aperture, seed germination and cotyledon greening assays. Finally, ABA-induced accumulation of SUMO-protein conjugates was reduced in *NSE2*/*MMS21* mutant. Altogether, this indicates that NSE2/MMS21 plays a role as negative regulator of ABA-mediated stress response, by SUMOylating ABA responsive gene products (or their transcriptional activators/repressors in a mechanism proposed above for FLC regulation) and thus influences stomata opening [112].

### 4.3. Suppressor of Immune Responses

Arabidopsis NONEXPRESSER OF PR GENES 1 (NPR1) is a key positive regulator of salicylic acid (SA)-mediated systemic acquired resistance (SAR) pathway essential for defence against microbial pathogens [113,114]. NPR1 function is critical for expression of PATHOGEN RESISTANCE (PR) genes. Among suppressors of *npr1* phenotype (i.e., PR genes are up-regulated), mutation in a gene named *SUPPRESSOR OF NPR1-1*, *INDUCIBLE* (*SNI1*) was identified [115]. Recently, purification of the SNI1 complex in Arabidopsis revealed that it interacts with an uncharacterized protein termed ARABIDOPSIS SNI1 ASSOCIATED PROTEIN 1 (ASAP1), SMC5 and SMC6B [42]. Although ASAP1 and SNI1 do not share significant sequence homology with any proteins outside of the plant kingdom, modelling of their structure revealed that they are structurally highly similar to the yeast NSE5 and NSE6, respectively (see Section *NSE5-NSE6* sub-complex for details). Hence, ASAP1 and SNI1 are the putative functional orthologues of yeast NSE5 and NSE6 in plants acting as suppressors of SAR by unknown mechanism(s). Screening for *SUPPRESSOR OF SNI* (*SSN*), i.e., for the mutations reverting smaller size *sni1* mutant plants to a wild-type like phenotype, revealed the following genes: *SSN1* (*RADIATION SENSITIVE 51D*), *SSN2* (*SWIM DOMAIN CONTAINING SRS2 INTERACTING PROTEIN 1*), *SSN3* (*BREAST CANCER 2A*), *SSN4* (*RADIATION SENSITIVE 17*) and *ATR* [42,94,116,117]. This suggests that either excessive production of SA and/or reduced genome stability in the absence of functional SMC5/6 complex lead to an increased frequency of repair via (possibly error prone) pathway represented by the *SSN* genes. After disrupting the function of the repair signalling components ATR and RAD17 and their putative downstream SSN effectors, the balance may be re-established, allowing for normal plant growth. 

## 5. Conclusions

Data from fungal, animal and plant models show that SMC5/6 complex operates within a large network controlling the maintenance of post-replicative chromosome structure. The key functions concentrate towards (i) removal of DNA topological stress in a process guided by cohesin complex and (ii) repair of particular types of DNA damage (most likely toxic replication intermediates). However, the SMC5/6 functions may be more diverse as indicated by several other examples mentioned in this review. Furthermore, surprising functions of the SMC5/6 complex are yet to be expected as indicated e.g., by the recent in vitro observation that it organizes microtubules into bundles or acts as a viral suppressor [118,119]. Large portion of the work remains to be done in understanding molecular functions of individual subunits and (often phylogenetic group specific) phenotypes of SMC5/6 complex mutants. In plants, this includes roles of SMC5/6 complex in abiotic and biotic stress responses, developmental control, gametogenesis and sporogenesis.

## Figures and Tables

**Table 1:** Overview of Structural maintenance of chromosomes (SMC) complex 5/6 subunits in budding yeast (*Saccharomyces cerevisiae*), fission yeast (*Schizosaccharomyces pombe*), fruit fly (*Drosophila melanogaster*), human (*Homo sapiens*) and Arabidopsis (*Arabidopsis thaliana*). NA-information not available.

*S. cerevisiae*	*S. pombe*	*D. megalonaster*	*H. sapiens*	*A. thaliana*
SMC5	SMC5/SPR18	SMC5	SMC5	SMC5
SMC6/RHC18	SMC6/RAD18	SMC6/JNJ	SMC6	SMC6A, SMC6B/MIM
NSE4/QRI2	NSE4/RAD62	NSE4	NSE4A, NSE4B	NSE4A, NSE4B
NSE1	NSE1	NSE1	NSE1	NSE1
NSE3	NSE3	NSE3/MAGE	NSE3/MAGE-G1	NSE3
NSE2/MMS21	NSE2/PLI2	QUIJOTE/CERVANTE	NSE2/MMS21	NSE2/MMS21/HPY2
NSE5/YML023c	NSE5	NA	SLF1	SNI1
KRE29	NSE6	NA	SLF2	ASAP1

**Table 2:** Overview of SMC5/6 complex subunits in plants. The species are represented by spreading earthmoss (*Physcomitrella patens*), *Brachypodium distachyon*, *Oryza sativa* (rice) and *Hordeum vulgare* (barley), *Solanum lycopersicum* (tomato) and *Arabidopsis thaliana* (Arabidopsis). The number of gene identifiers indicates the number of copies per genome. * Functional (not protein sequence-based) homologs. Letters next to *S. lycopersicum* genes indicate transcript [94] in roots (R), leaves (L), flower buds (Fb), open flowers (Fl) and fruits (Fr). Transcriptional data for Arabidopsis are provided in Figure 2A. Genes for *P. patens*, *B. distachyon*, *O. sativa and H. vulgare* were identified by BLAST searches in Phytozome, for tomato in the Sol Genomics Network database (https://solgenomics.net/) and for Arabidopsis in TAIR (https://www.arabidopsis.org/index.jsp).

Subunit	*P. patens*	*B. distachyon*	*O. sativa*	*H. vulgare*	*S. lycopersicum*		*A. thaliana*
*SMC5*	*PpSMC5*	*BdSMC5*	*OsSMC5*	*HvSMC5*	*SlSMC5*		*AtSMC5*
	Pp3c24_4940	Bradi2g14160	LOC_Os05g51790	HORVU1Hr1G095230	Solyc01g087720	L, Fr	At5g15920
*SMC6*	*PpSMC6*	*BdSMC6*	*OsSMC6*	*HvSMC6*	*SlSMC6*		*AtSMC6A*
	Pp3c11_11190	Bradi4g08527	LOC_Os09g03370	HORVU5Hr1G050720	Solyc05g051680	R, L, Fl, Fr	At5g07660
							*AtSMC6B (MIM)*
							At5g61460
*NSE1*	*PpNSE1*	*BdNSE1*	*OsNSE1*	*HvNSE1*	*SlNSE1*		*AtNSE1*
	Pp3c20_10070	Bradi4g43810	LOC_Os12g03360	HORVU0Hr1G010660	Solyc01g006210	R, L, Fl, Fr	AT5G21140
		Bradi2g12255	LOC_Os11g03590				
*NSE2*	*PpNSE2*	*BdNSE2*	*OsNSE2*	*HvNSE2*	*SlNSE2*		*AtNSE2/MMS21/HPY2*
	Pp3c22_18560	Bradi2g16600	LOC_Os05g48880	HORVU1Hr1G087520	Solyc07g062780	R, L, Fl, Fr	
		Bradi2g16580					At3g15150
*NSE3*	*PpNSE3A*	*BdNSE3*	*OsNSE3*	*HvNSE3*	*SlNSE3*		*NSE3*
	Pp3c15_18480	Bradi1g58440	LOC_Os07g05650	HORVU2Hr1G060140	Solyc10g018870	R, L, Fl, Fr	At1g34770
*NSE4*	*PpNSE4*	*BdNSE4*	*OsNSE4*	*HvNSE4*	*SlNSE4A*		*NSE4A*
	Pp3c27_130	Bradi3g06970	LOC_Os02g10090	HORVU7Hr1G094270	Solyc10g078730	R, L, Fl, Fr	AT1G51130
		Bradi1g35930	LOC_Os06g41380	HORVU6Hr1G033750	Solyc12g041890	Fb	*NSE4B*
			LOC_Os08g40010		Solyc01g006460	Fb	At3g20760
			LOC_Os02g29620		Solyc04g025510	L, Fl buds	
			LOC_Os04g10870				
			LOC_Os07g01010				
NSE5*	*PpASAP1*	*BdASAP1*	*OsASAP1*	*HvASAP1*	*SlASAP1*		*ASAP1*
	Pp3c4_7040	Bradi2g08380	LOC_Os01g13940	HORVU3Hr1G032750	Solyc11g066340	R, L, Fl, Fr	At2g28130
NSE6*	*PpSNI1*	*BdSNI1*	*OsSNI1*	*HvSNI1*	*SlSNI1*		*SNI1*
	Pp3c13_1090	Bradi3g11450	LOC_Os02g20870	HORVU6Hr1G054340	Solyc02g077320	R, L, Fl, Fr	At4g18470
